# mRNA Delivery: Challenges and Advances through Polymeric Soft Nanoparticles

**DOI:** 10.3390/ijms25031739

**Published:** 2024-02-01

**Authors:** Samaneh Yousefi Adlsadabad, John W. Hanrahan, Ashok Kakkar

**Affiliations:** 1 Department of Chemistry, McGill University, 801 Sherbrooke St West, Montreal, QC H3A 0B8, Canada; samaneh.yousefiadlsadabad@mail.mcgill.ca; 2 Department of Physiology, McGill University, 3655 Promenade Sir-William-Osler, Montreal, QC H3G 1Y6, Canada; john.hanrahan@mcgill.ca

**Keywords:** mRNA therapeutics, cationic and non-cationic polymers, polymer lipid hybrid systems

## Abstract

Single-stranded messenger ribonucleic acid (mRNA) plays a pivotal role in transferring genetic information, and tremendous effort has been devoted over the years to utilize its transcription efficacy in therapeutic interventions for a variety of diseases with high morbidity and mortality. Lipid nanocarriers have been extensively investigated for mRNA delivery and enabled the rapid and successful development of mRNA vaccines against SARS-CoV-2. Some constraints of lipid nanocarriers have encouraged the development of alternative delivery systems, such as polymer-based soft nanoparticles, which offer a modular gene delivery platform. Such macromolecule-based nanocarriers can be synthetically articulated for tailored parameters including mRNA protection, loading efficacy, and targeted release. In this review, we highlight recent advances in the development of polymeric architectures for mRNA delivery, their limitations, and the challenges that still exist, with the aim of expediting further research and the clinical translation of such formulations.

## 1. Introduction

Genetic therapies are rapidly gaining momentum as highly effective therapeutic interventions for many diseases. Gene addition strategies in which nucleic acid constructs that encode a therapeutic gene are delivered as DNA (deoxyribonucleic acid) or chemically modified mRNA (messenger RNA) critically depend on efficient delivery. Gene editing approaches involving CRISPR-Cas9, base editing, super-exon insertion, prime editing, anti-codon edited tRNA (transfer RNA), and antisense oligonucleotide to repair defects in endogenous genes similarly depend on the efficient delivery of nucleic acids to target cells [1,2,3,4,5]. Nucleic acid-based therapeutics that enable cells to produce proteins help to resolve key issues related to the use of exogenous proteins including isolation/purification, high synthetic costs, limited solubility, unsuitable glycosylation, and cell targeting [6]. The recent development of mRNA vaccines for COVID-19 has demonstrated the benefits of nucleic acid delivery, notably that of single-stranded mRNA, as an efficacious treatment platform [7]. mRNA mediates protein expression in cells and offers a broad range of biomedical applications such as genome editing [8], cancer immunotherapy [9,10,11], vaccines [12,13,14], tissue regeneration [15], protein replacement therapies [16], and cellular reprogramming [17], etc. mRNA has several advantages over DNA as follows: (i) it only needs to be inserted into the cytoplasm to be expressed, whereas DNA must overcome the nucleus barrier for its function [18]; (ii) it is safer as mRNA does not enter the cell nucleus, so it cannot cause mutations [19]; (iii) tremendous research has led to low-cost mRNA synthesis through the in vitro transcription of mRNA using a template and established purification processes [20]; and (iv) mRNA enables fast protein expression and has a relatively short half-life, which minimizes its expression in other cells and off-target effects that may occur with DNA [6,20]. 

Despite many potential advantages of mRNA over DNA, the clinical translation of mRNA-based therapies has been challenging because (i) it is a large biomolecule with a net negative charge, which hampers cellular uptake due to electrostatic repulsive forces with the cell membrane [21]; (ii) it has a single-stranded structure, which makes it fragile, and is rapidly degraded by ribonucleases present in the extracellular matrix [22]; and (iii) many of the mRNA molecules taken up by cells undergo endosomal degradation before reaching the cytoplasm [13,23]. Stability of mRNA, which in turn influences its translation efficacy, can be enhanced through the modification of its five structural components as follows: 5′ cap, 3′ poly A tail, the 5′ and 3′ untranslated regions, and the translatable region. Some examples of such chemically modified nucleosides include N1-methyl pseudouridine in place of uridine [24], 5′ caps [25], and the optimization of the poly A tail length named [26]. These stability issues in addition to the two above-mentioned constraints emphasize the need to engineer mRNA delivery systems. The design of circular mRNA that resists degradation, the integration of a modified nucleoside into the structure of mRNA, and the purification of synthetic mRNA using HPLC instead of precipitation are some other examples of chemical engineering of mRNA [27]. 

Viral [28] and non-viral [29,30,31] vectors are common strategies for delivering nucleic acids to desired sites. However, viral delivery has limitations due to (i) inherent toxicity, (ii) triggering immune responses [1], and (iii) high costs due to difficulty in scaling up production [32]. There has been extensive effort devoted to developing non-viral vectors, especially those based on liposomes, lipid nanoparticles, polypeptides, protamines, polymers, micelles, inorganic-based materials, and hybrid systems. Amongst these, liposomes, and vesicle-type lipid structures with a hydrophilic core have been most extensively investigated and have contributed towards several successful clinical translations. Liposomes formed from biocompatible molecules offer protection from ribonucleases and allow for efficient cellular uptake due in part to their resemblance to the cell membrane [33,34]. There are four principal components involved in the structural build-up of liposomes, which include helper phospholipids (leading to increased stability and fluidity of liposome), PEG-lipid (PEG = polyethylene glycol, used for enhanced in vivo circulation time and stability), cationic lipid (increasing mRNA encapsulation efficiency through electrostatically binding with mRNA), and cholesterol (manipulating liposomal permeability) [35]. Liposome-based technology was utilized in the development of COVID-19 vaccines.

**Figure 1:**
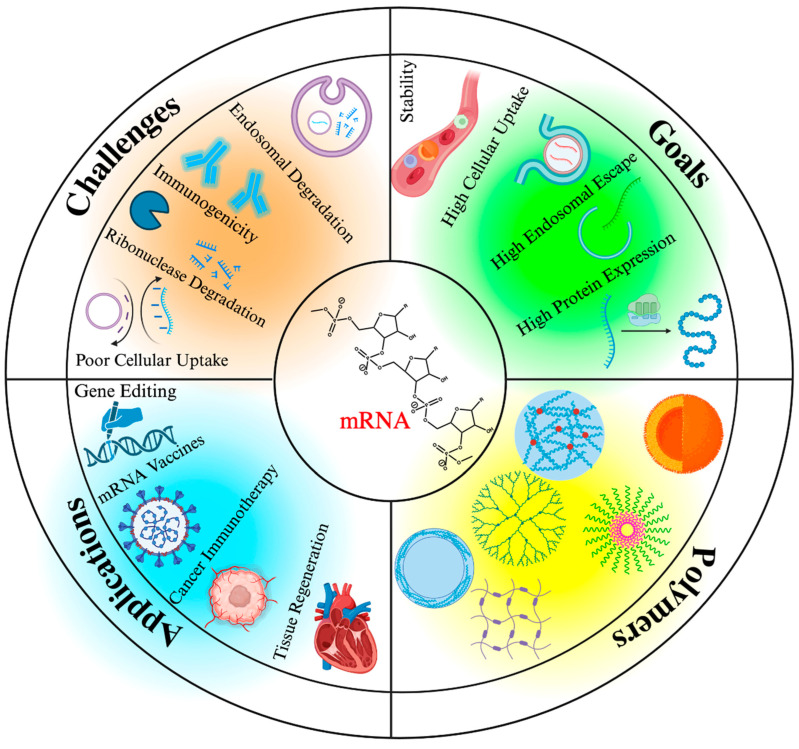
mRNA delivery: Challenges, goals, widely explored polymer-based systems, and intended applications of this technology. Created using BioRender, www.biorender.com (accessed on 12 November 2023).

Despite substantial achievements, lipid nanoparticles suffer from low stability. Their disassociation in biological media can lead to off-target delivery and insufficient loading of the target cells [36]. In addition, there are constraints on the structural diversity of lipid nanoparticles that may limit the design of functionalized lipids and cargo loading efficiency [37]. This emphasizes the need to develop alternative carriers that may address these crucial issues by enabling structurally optimized formulations for maximal encapsulation efficiency and endosomal escape and hence enhanced transfection efficiency. Polymers with tremendous versatility in structure, composition, architectural complexity, and synthetic articulation through the addition of functional groups and surface modifications (Figure 1) offer a versatile platform for this purpose. Advances in synthetic polymer chemistry have facilitated the design of well-defined polymeric structures with predetermined properties [38,39]. Here, we discuss the current state of the art in polymeric architectures when designing nanocarriers for mRNA delivery, their advantages and limitations, and the future outlook for the delivery of mRNA and other macromolecules.

### Polymeric Architectures

Macromolecules with varied compositions, morphology (linear, branched), molecular weight, crosslinking density, and end/backbone functionalities continue to be at the forefront of research. The resulting polymer diversity is being utilized in designing nanomaterials for applications in drug delivery [40], tissue engineering [41], self-healing wound–burn dressings [42,43], contact lenses [44], and dentistry [45,46]. A major advantage of polymers is the tunability of their physical and chemical properties through structural manipulation and the introduction of desired functional groups. Soft nanoparticles from amphiphilic block copolymers have been extensively investigated for small molecule delivery [47,48,49]. Their utility provides the impetus for the similar optimization of polymeric formulations for mRNA. In general, polymeric architectures for mRNA delivery can be categorized as cationic, non-cationic, and stimuli-responsive polymers. A brief overview of the methods employed for mRNA complexation in polymers is given below. It is then followed by a discussion of the polymers utilized in each category and the efficacy of their design parameters in mRNA delivery.

## 2. Preparation of mRNA-Polyplex Nanoformulations

The methodologies used to assemble mRNA-loaded nanoparticles are as important as the physicochemical properties of polymers since these determine the overall size, morphology, stability, and surface characteristics of nanoparticles. An understanding of the procedural details can help determine which method may be better suited for a particular application (Table 1).

### 2.1. Direct Mixing

Mixing is the most commonly used method for preparing mRNA-loaded nanoparticles. It involves dissolving polymeric formulations in an organic solvent and mRNA in aqueous medium separately, then mixing them together using a hand-held pipette, a jet mixer, or a microfluidic device. The solubility of the desired polymer in a small amount of water-miscible organic solvent (usually DMSO (dimethylsulfoxide) or ethanol are commonly employed for the purpose) is important. It is then added to aqueous mRNA solution [50,51,52]. A recent study examined the efficacy of this mixing method in delivering mRNA, which was loaded into lipid nanoparticles. A solution of the desired compositions containing lipid, cholesterol, 1,2-dioleoyl-sn-glycero-3-phosphoethanolamine (DOPE), and PEG2000 was prepared in ethanol containing 10% pH 4 citrate buffer. It was either added manually or through microfluidics to an aqueous solution of mRNA. Efficacy of the nanoparticles in vivo depends on the method used to prepare them. Properties of nanoparticles prepared using microfluidics depend on the microfluidic mixing parameters, and one study concluded that each formulation needs to be optimized separately in terms of these mixing parameters used [53].

**Table 1:** Comparative analysis of the varied methodologies used to assemble mRNA-loaded nanoparticles.

Method	Advantages	Disadvantages	Reference
Direct Mixing: (i) Hand mixing (ii) Jet mixer/microfluidics	(i) Low-cost, easy. (ii) Controlled mixing, high reproducibility, scalability, and consistent nanoformulations with high encapsulation efficiency.	(i) Uncontrolled mixing, poor reproducibility, and low to moderate encapsulation efficiency. (ii) High-cost, using large amounts of organic solvents.	[54]
Double emulsion/solvent evaporation	Simple, control over varied parameters, high encapsulation efficiency (especially for water-soluble cargos).	Difficult to control particle size as well as polydispersity and not easy to scale up.	[55]
Salting out	High reproducibility, avoids the use of chlorinated solvents, suitable for loading proteins and nucleic acids, no need for heating.	Low encapsulation efficiency for hydrophilic cargos, low scalability, time-consuming, needs the vigorous homogenizing instrument.	[56]
Nanoprecipitation	Easy, high reproducibility, controlled size, scalable.	Low encapsulation efficiency, high dependency of nanoparticle diameter to the stir rate.	[57]
Flash Nano-complexation	Organic solvent-free, fast, controlled particle size, scalable, and high encapsulation efficiency.	Requires control over electrostatic interactions (N:P ratio).	[58]

### 2.2. Double Emulsion/Solvent Evaporation

**Figure 2:**
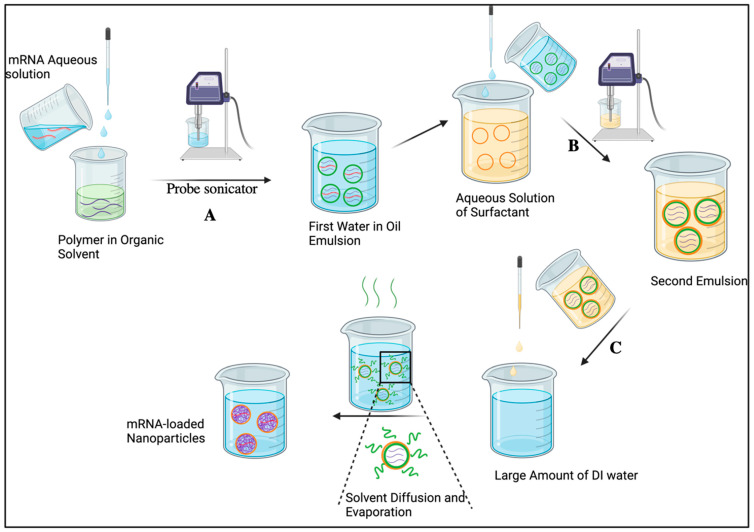
Double emulsion/solvent evaporation method: Step A, formation of the first water-in-oil emulsion; Step B, addition to a solution of a surfactant yielding the second emulsion; Step C, diffusion of the organic solvent into the aqueous phase followed by organic solvent evaporation. Created using BioRender, www.biorender.com (accessed on 14 November 2023).

This method, as the name suggests, involves preparing an emulsion in two steps and then evaporating the organic solvent to form nanoparticles (Figure 2). The polymer is dissolved in a water-immiscible, low-boiling solvent (dichloromethane, chloroform, or ethyl acetate) and subsequently mixed with the aqueous phase containing mRNA. The first emulsion is obtained by vigorous stirring or using probe sonication (Step A, Figure 2). It is then added to an aqueous solution containing a surfactant (polyvinyl acetate, vitamin E, tocopheryl polyethylene glycol succinate (TPGS), or various alkylated polyethylene glycol (PEG) chains), which is followed by emulsification to form the second emulsion (Step B). The latter is subsequently added to a large volume of water to induce diffusion of the organic solvent into the aqueous phase (Step C), which is followed by the removal of organic solvent through evaporation [59]. Many parameters can affect the physical properties of the nanoparticles formulated using this method, including emulsion formation, the amplitude of sonicating or stirring, ratio of constituents, and the amount of surfactant used, etc. [60].

A single emulsion method, in which polymer and mRNA are dissolved in an organic solvent and then added to an aqueous phase followed by emulsification, has also been developed for mRNA loading [59]. However, this technique is rarely used due to the poor solubility of mRNA in organic solvents. To circumvent the solubility problem, one can prepare nanoparticles separately and then load them by incubation in an mRNA solution [61].

### 2.3. Salting-Out

**Figure 3:**
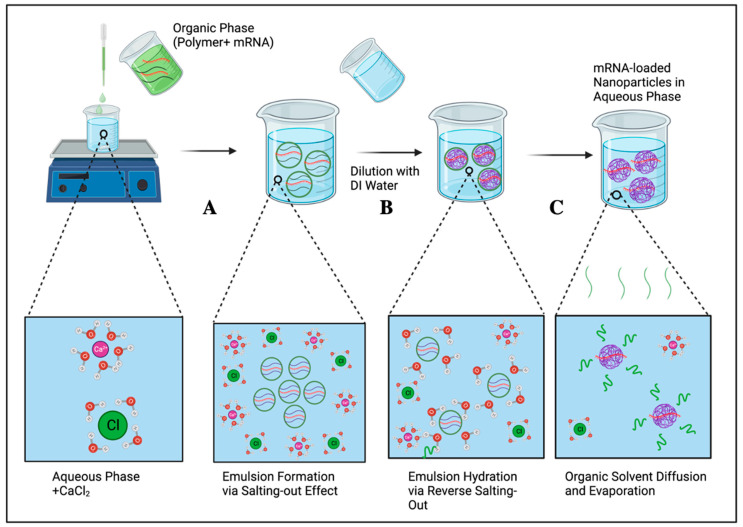
Salting-out method: A, emulsion formation by salting-out; B, dilution with water; C, diffusion of organic solvent into water followed by evaporation of the organic solvent. Created using BioRender, www.biorender.com (accessed on 14 November 2023).

In this method, an emulsion of an aqueous phase and water-miscible organic solvent (acetone or ethanol) is prepared by reducing the solubility of the solute in water by the addition of MgCl_2_ and CaCl_2_, which compete with the solute for interactions with water molecules (Figure 3). It involves emulsifying the organic phase containing polymer and mRNA with an aqueous phase which contains a high concentration of the salt. The emulsion is subsequently diluted with distilled water to reverse the salting-out effect. A brief description of this method is presented in Figure 3 as follows: (i) Step A, a solution of the polymer and mRNA in a water-miscible solvent is added dropwise to an aqueous phase containing the desired salt, which is followed by stirring. mRNA is emulsified due to the higher tendency of the water molecules to associate with calcium/magnesium and chloride ions (salting-out); (ii) Step B, the emulsion is subsequently diluted with water to increase the hydration of the mRNA/polymer emulsion (reverse salting-out); (iii) Step C, this leads to the enhanced diffusion of the organic phase into the aqueous phase, which is followed by the evaporation of the organic solvent and formation of mRNA-loaded nanocarriers. The formulated nanoparticles are then purified from the salt solution through filtration [62,63]. Although this technique does not require the use of chlorinated solvents and is appropriate for loading biotherapeutics such as DNA, RNA and proteins, lower encapsulation efficiency of hydrophilic cargo compared with other methods (Table 1) has prevented its widespread use [64,65].

### 2.4. Nanoprecipitation

In this method, polymeric precursors are first dissolved in polar organic solvents such as dimethyl sulfoxide (DMSO), ethanol, tetrahydrofuran (THF), or dimethylformamide (DMF), and then mixed with mRNA buffer solution. The mixture is then dialyzed against water to remove the organic solvent through the passive diffusion. The polymer/mRNA solution can also be added directly into a large volume of water dropwise without dialysis, leading to precipitation as the organic solvent diffuses into water [66]. Nanoprecipitation is simple, scalable, and reproducible (Table 1); however, the encapsulation efficiency of mRNA and other hydrophilic cargos is low [67]. For example, the encapsulation efficiency of ciprofloxacin, a hydrophilic drug, into poly (D, L-lactic acid)-dextran and PLGA-PEG-based nanoparticles prepared using the nanoprecipitation method was found be ~1.50 and 1.86%, respectively [68].

### 2.5. Flash Nano-Complexation

**Figure 4:**
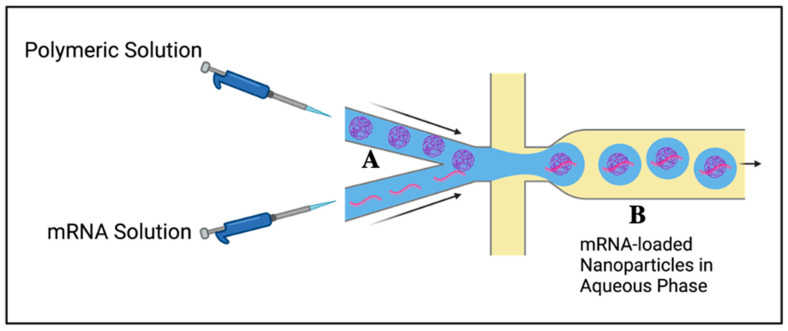
Flash nanocomplexation method: A, mixing of polymer and mRNA solutions through controlled jet streams; B, formation of mRNA-loaded nanoparticles. Created using BioRender, www.biorender.com (accessed on 12 November 2023).

This technique differs from the methodologies described above and has several advantages as follows: (i) It does not require the use of organic solvents that could harm the environment or have toxicity; therefore, it is of great value in biomedical applications. Although organic solvents can generally be removed from nanoparticles, nanoparticles may still contain trace amounts; (ii) it uses both covalent and non-covalent interactions between polymeric systems and mRNA (leading to nanoparticles with higher stability) [69] rather than only relying on hydrophobic interactions and solvent displacement to drive the production of nanoformulations; and (iii) it utilizes a controlled jet mixer to combine fixed amounts of solution at specific rates of addition, leading to nanoformulation uniformity (Figure 4). Electrostatic interaction between the positively charged polymers (N) and negatively charged mRNA (P) produces mRNA-loaded nanocomplexes [70]. It has been suggested that the N:P ratio can play a key role in the overall size, encapsulation efficiency, stability and cellular uptake of mRNA-loaded nanoparticles [71]. To demonstrate this effect, three different formulations of lipids containing cationic amines and mRNA with N:P ratios of 4:1, 6:1, and 8:1, respectively were prepared. It was noted that the nanoparticles from the lowest N:P ratio (4:1) were larger (~115 nm) compared with those with ratios of 6:1 and 8:1 (~90 nm). The encapsulation efficiency (%) was found to be slightly lower in larger nanoparticles (4:1 N:P ratio, ~88%) than in the smaller ones (6:1 and 8:1 N:P ratio, ~96%) [72].

Several techniques have been employed for preparing mRNA-loaded polymeric nanoformulations, but the most extensively used one (Table 2) is that of direct mixing mRNA solution with a diluted solution of the polymer in acidic buffer [73]. Mixing can be done through hand pipetting, using microfluidic devices with controlled rates of injecting the two phases, or using a jet mixer that provides nanoparticles with uniform hydrodynamic diameter and morphology. For instance, PBAE-co-PCL terpolymer with the molecular weight of mRNA-interacting polymer (PBAE) in the range of 850–2800 Da was loaded with mRNA using the direct mixing method. The polymer was initially dissolved in a small amount of DMSO and then diluted in sodium acetate buffer. Nanoparticles formed by directly mixing of both mRNA and polymer solutions in buffer were characterized using DLS (dynamic light scattering), zeta potential measurement, and cryo-TEM (cryo-transmission electron microscopy). Due to the poor solubility of polymers in an aqueous medium, small nanoparticles were obtained. These nanoparticles had lower transfection efficiency compared with the ones that were prepared using a premixing method (dissolution of the polymer in ethanol first and then mixed with mRNA) [71]. It suggests that each methodology used in preparing formulations (Table 1 and Table 2) has its own advantages and disadvantages. Optimizing the technique and finding mRNA-compatible organic solvents continues to play a key role and is an area of intense investigation. In addition to nanoparticle preparation methodologies, their characterization using DLS, zeta potential, and TEM is essential. DLS and zeta potential can help determine mean diameter and surface charge of nanoparticles, respectively [74]. However, these do not provide accurate data related to different populations of nanoparticles. TEM can give an estimation of the overall morphology and size of the nanoparticles. The TEM measurement generally requires several steps, including sample preparation, staining, and drying of the sample on a grid. Each of these may lead to variability in TEM results. It is generally essential to characterize nanoformulations using a combination of techniques to obtain a clear picture of their properties [75].

## 3. Polymeric Architectures for mRNA Delivery

### 3.1. Cationic Polymers

**Figure 5:**
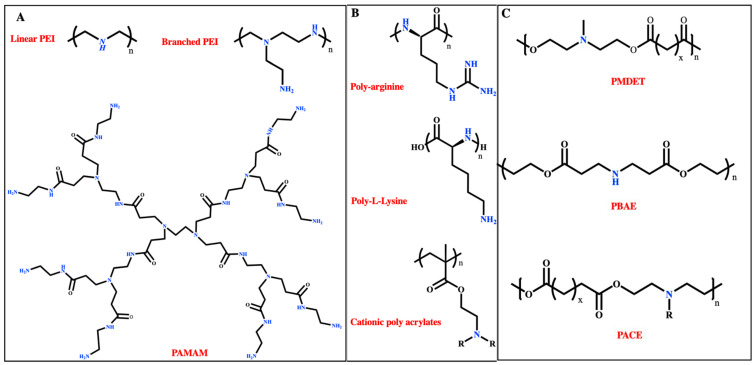
Chemical structures of cationic polymers used for mRNA delivery: (**A**) linear and branched polyethylene imine (PEI) and polyamidoamine (PAMAM); (**B**) poly-arginine and poly-l-lysine, and cationic polyacrylates; (**C**) poly (β-amino esters) (PABE), poly (N-methyl diethanol amine) (PMDET), and poly(amino-co-ester) (PACE).

Considering the inherent negative charge of mRNA, polymers that contain positively charged moieties and interact electrostatically with mRNA have been extensively studied for their ability to increase mRNA loading efficiency [97,98]. Such polymers contain primary, secondary, or tertiary amines in their backbones (Figure 5). Some examples of this class of polymers are described below along with a discussion of recent developments [99].

#### 3.1.1. Polyethyleneimine (PEI)

**Figure 6:**
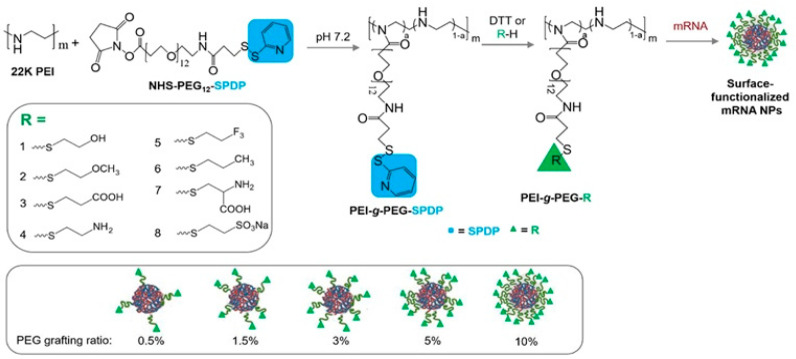
Different end group-functionalized PEG chains grafted on PEI, thus leading to surface-functionalized nanoparticles: synthesis through the grafting of PEG-SPDP onto PEI in varied ratios followed by the exchange of the SPDP group with small, functionalized thiol molecules (R1-R8); self-assembly and loading of mRNA. Reproduced with permission from [70].

Linear and branched polyethyleneimine are the earliest examples of cationic polymers used as mRNA vectors. They have several advantageous characteristics including (i) a high density of positive charge due to the presence of multiple amine centers separated by short alkyl chains [100]; (ii) high loading efficiency (>70%) [101]; (iii) increased cellular uptake mediated by electrostatic attraction with the negatively charged cell membrane; and (iv) endosomal escape that is induced by the “sponge effect” in which the protonation of amine groups increases the entry of anions and water molecules into endosomes. Branched PEIs have amines with a wide range of pK_a_ values and variable degrees of protonation that can be used to tailor mRNA loading and transfection efficiency [102]. However, the positive charge that promotes interaction with mRNA has also hindered clinical translation, as it leads to immunogenicity and the adsorption of negatively charged serum proteins on the nanoparticle surface. This issue is being addressed by modifying PEIs with biocompatible moieties that help to conceal positively charged centers and prolong in vivo circulation times. PEGylation is the most common approach for this purpose as PEG is biocompatible, soluble in biological fluids, and serves as a protective layer that prevents serum protein adsorption on the surface. For example, different ratios of PEG chains were grafted onto the functionalized PEI end groups in preparing nanocarriers for the delivery of mRNA to lung cells (Figure 6). To synthesize polymeric precursors, an NHS-PEG-SPDP chain was grafted onto the PEI by reacting its amine groups with the NHS (N-hydroxy succinimide) ester group of PEG. Small molecules containing a thiol group were replaced with SPDP (pyridyl disulfide) by thiol–disulfide exchange, resulting in the core–shell type of nanoparticles with varied percentages of PEG shells functionalized with different end groups that protected the PEI-based core and mRNA. It was noted that (i) increasing the PEG percentage from 0.5 to 10% led to a decrease in transfection efficiency (~4 times); (ii) nanoparticles with the grafted PEG ratio of 0.5% were found to have higher mRNA transfection efficiency in lungs than in other organs including the heart, spleen, liver, and kidney; and (iii) nanoparticles with 0.5% PEG containing amine and amino acid end groups showed 5–10 times higher transfection efficiency in lungs than polymeric counterparts containing other end groups [70]. Since PEG chains could hinder mRNA-PEI interactions, the above strategy has its limitations, as high molecular weight PEG and PEG/PEI ratios can impede the critical role of PEI chains in mRNA transfection efficiency. This suggests that an appropriate balance between PEG and PEI chain ratios is essential for optimal efficiency [70]. More recently, low molecular weight PEG chains have been employed in the design of PEI-based mRNA vectors.

Introducing biodegradable polymeric segments into PEI-based nanocarriers has also been investigated. Nanoparticles were prepared from poly (lactide-co-glycolide) and PEI for the delivery of mRNA, encoding a green fluorescent protein, into human monocyte-derived dendritic cells. The inclusion of biodegradable esters did not result in any noticeable toxicity [101]. Fluoroalkane-grafted PEI-based vectors have also been designed for the development of mRNA vaccines. The latter were taken up by dendritic cells, which caused the stimulation of the immune system in fighting against the cancer antigen [76]. Some other examples of PEI hybrid systems used for mRNA delivery include PLGA/PEI [101,102], vitamin E succinate-modified PEI [103], graphene quantum dots functionalized with PEI [104], and the conjugation of PEI with β-cyclodextrin [77].

#### 3.1.2. Poly (β-Amino Esters)

Poly (β-amino esters) (PBAE) can be easily synthesized using a simple Michael addition reaction of amines and di-acrylates that leads to a step-growth polymerization with no by-products. The synthetic procedure can be adapted to include a wide variety of monomers, and the resulting polymers can thus have diverse backbones, side chains, and functional end groups, allowing one to tailor the composition for specific purposes [50]. For example, a variety of PBAEs were synthesized with oligopeptide end groups to (i) enhance their hydrophilicity; (ii) avoid non-specific accumulation of the nanoparticle formulation in the liver; and (iii) target antigen-presenting cells and initiate an immune response [79]. In addition to the presence of amine groups that provide a strong sponge effect, PBAEs are biodegradable, as the ester bonds in their backbone can be hydrolysed to generate non-toxic β-amino acids [105].

**Figure 7:**
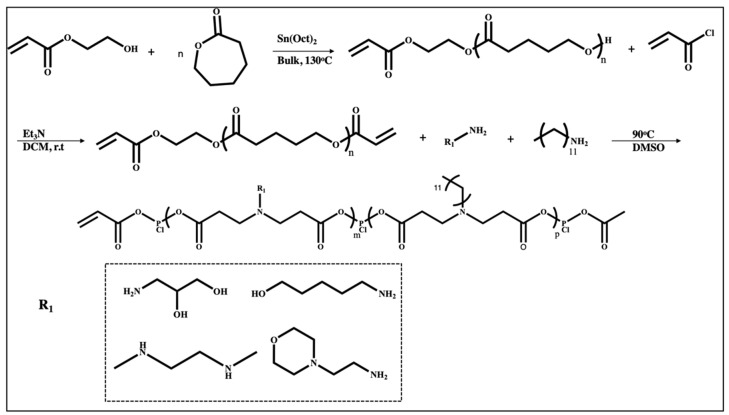
Terpolymer synthesis based on ring opening polymerization of caprolactone in the first step followed by Michael addition with different amines containing varied R_1_ groups [78].

Biodegradability may limit the cytotoxicity caused by positive charge, and PBAE has attracted more attention than PEIs. For example, PBAE-co-PCL based terpolymers were prepared as mRNA vectors, and their delivery efficacy was evaluated in vivo and in vitro. Terpolymer synthetic methodology involved the synthesis of polycaprolactone-based diacrylates with a different number of caprolactone units followed by Michael addition with a variety of substituted primary amines (Figure 7). More specifically, hydroxy ethyl acrylate was polymerized with caprolactone through the ring-opening polymerization, which was followed by the end group modification of polycaprolactone with acryloyl chloride to form diacrylate-polycaprolactone. In the next step, the desired amine was reacted with diacrylate-based polycaprolactone through Michael addition. The transfection efficiency with such polymers was highly dependent on the lipophilicity of the polymeric segments and the number of caprolactone chains incorporated into the structure. Increasing the number of polycaprolactone chains to five in a terpolymer with 5-amino-1-pentanol on both ends led to a 250-fold increase in transfection efficiency. However, a further increase in hydrophobicity reduced the positive charge density that is crucial for mRNA loading [78].

There is a direct relationship between PBAE molecular weight and cytotoxicity. The higher the polymer molecular weight, the slower its degradation and the greater its toxicity. A higher PBAE molecular weight enables strong interaction with mRNA and efficient mRNA loading. Branched PBAEs with molecular weights > 25 kDa have high transfection efficiency and toxicity, while those with molecular weights ≤ 1.8 kDa are less toxic and non-transfecting [106]. This issue has been addressed by introducing quaternized amines into the low molecular weight PBAE to enhance binding and lowering the toxicity of the resulting mRNA polyplexes [59].

Most polymeric structures used to deliver nucleic acids have a linear structure, and studies utilizing branched polymers are rare. Considering the many advantages offered by branched polymers compared with their linear analogues having same composition [107], branched polymeric systems for mRNA delivery are expected to have higher efficacy. Branched PBAE is less toxic than its linear counterparts of similar molecular weights, which may be attributed, at least in part, to its complex architecture, different composition, and multi-tasking end groups [108]. Hyperbranched PBAE-based nanoformulations were prepared to deliver luciferase mRNA to lung cells through inhalation. Luciferase was expressed in the lung 24 h-post inhalation, and there were no signs of mRNA distribution to other cells [80]. An injectable wound-healing hydrogel had also been developed that was loaded with the mRNA-PBAE polyplex to produce a biocompatible vector with sustained polyplex release [51]. Lipid-modified PBAE has also been explored with the goal of increasing hydrophobicity. Lipid-modified PBAE enhances the stability of mRNA encoding spike protein in aqueous solution, and the vaccine can be stored at −20 °C for 12 months with no changes in the physiochemical properties of the polyplexes. Lipid-modified particles were found to have higher transfection efficiency (up to ~7-fold) compared with that of unmodified systems [61].

#### 3.1.3. N-Methyl Diethanol Amine (MDET)-Based Polyesters

**Figure 8:**
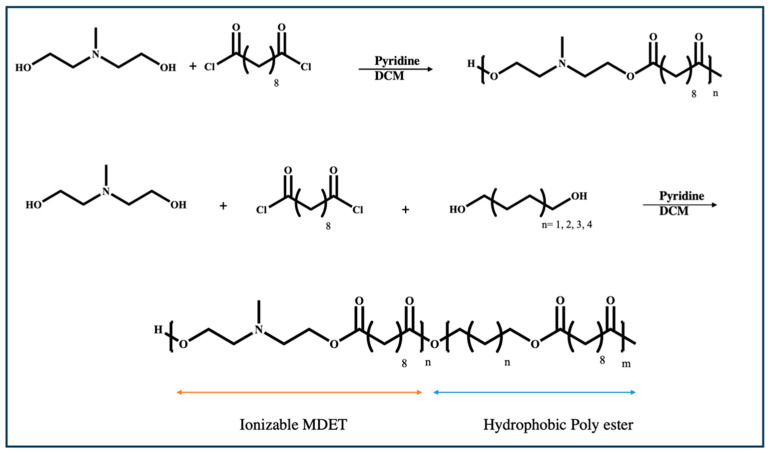
Synthetic scheme for poly (MDET) and poly (MDET-co-polyester) using the condensation of dialcohols with sebacoyl chloride [82].

MDET-based polyesters have similar functional groups to those in β-amino esters and additionally contain ionizable amine groups that reduce the need for adjuvants in ester-based biocompatible vectors. Two series of MDET-based polyesters for mRNA delivery have been prepared through the condensation of different alkyl-diols with sebacoyl chloride and N-methyl diethanol amine (Figure 8) [82]. In the first one, N-methyl diethanol amine was reacted with sabacoyl chloride, whereas in the second one, in addition to the aforementioned reagents, alkyl diols with varied chain lengths were introduced into the structure of polyester to impart hydrophobicity to the polymer. This led to the synthesis of a polyester with ionizable MDET and hydrophobic blocks in a one-pot esterification reaction.

PMDET-C4 and PMDET-C6 polymers had the highest transfection efficiency into lung and dendritic cells (~10-fold higher than that of PEI30 and jetOPTIMUS), without inducing any significant inflammatory response. Delivery of these polyplexes to lungs was achieved without using any targeting moieties [82].

#### 3.1.4. Poly (Amidoamine)

Hyperbranched and monodisperse macromolecules, referred to as dendrimers, have also been investigated for gene delivery. Dendrimers offer an ordered structure with several branched arms emanating from the core. Their high surface functionality and monodisperse nature distinguish them from linear polymers and make them attractive carriers for gene delivery [109,110,111]. Polyethyleneimine (PEI), poly(amidoamine) (PAMAM), poly(N-isopropylacrylamide) (PNIPAM), and polypropylene imine (PPI) are some of the most extensively investigated examples of dendrimers as nanocarriers. PAMAM [112] dendrimers contain several amide and tertiary amine groups inside the backbone and have multiple primary amine groups on their surface [113]. The latter lead to very high mRNA loading efficiencies and promote the sponge effect in endosomes.

PAMAM dendrimers can be prepared using a cost-effective two-step synthesis wherein aliphatic chains containing amine groups acting as core units, react with methyl methacrylate through Michael addition, which is followed by the amidation of esters with ethylene diamine [114,115]. The repetitive, layer-by-layer build-up leads to the formation of different generations and an exponential growth in the number of surface amine groups. One could design a specific generation, modify the surface primary amines, and control various parameters for cellular uptake, endosomal escape, drug loading efficiency, and the targeting of specific cells and organs. Despite being biodegradable, PAMAM dendrimers have high toxicity due to the high density of positively charged amines on their surfaces, and they are degraded slowly. PEGylation can help reduce the exposure of the positively charged surface, but it inhibits cellular uptake. This suggests the need to develop PAMAM-based polymers that have specific ligands on their surface, which could target them to cells. PAMAM-PEG dendrimers with galactose as a targeting moiety that specifically delivers mRNA to hepatocytes in the liver have been developed [116]. PAMAM-based vaccines have been developed as the first non-viral vectors for self-amplifying mRNA, which are efficient against three types of viruses (H1N1 influenza, Ebola, and Toxoplasma gondii. PAMAM dendrimers were modified with 2-tridecyl oxirane and subsequently combined with lipid-attached PEG for the fabrication of dendrimer-based nanoparticles. The latter were used to deliver antigen-coding mRNA and elicit the production of the antibody and CD8^+^ T cell-driven response. These nanoparticles had considerable mRNA expression efficiency in a wide variety of cell lines [86].

#### 3.1.5. Poly Amino Co-Ester

Poly amino-co ester (PACE) is a terpolymer class formed through the enzymatic copolymerization of lactones, diacids or diesters, and amine-substituted diols. Each segment serves a specific purpose. Esters induce hydrophobicity and biodegradability, and there is less positive charge density. Polyamino esters address some of the common issues associated with more extensively used PEIs in mRNA delivery. For example, utilizing low molecular weight polymer chains controls the positive charge density and helps reduce toxicity. In addition, amine diols introduce a moderate cationic charge, which facilitates cargo release when compared to highly charged vectors. The association of long chain diacids or diesters can provide control over hydrophobicity and end group conjugation [117]. Subtle changes in the structure of each fragment can have a huge impact on the type of nucleic acid that is to be delivered and the transfection efficiency. For example, three different PACEs in two different formulations (polyplexes and nanoparticles) were prepared for delivering pDNA, siRNA, and mRNA by introducing small changes in the structure of PACE. The general synthetic procedure involved the lipase-catalysed copolymerization of a lactone with N-methyl diethanol amine (MDEA) and a diester or a diacid. Poly(pentadecalactone-co-N-methyldiethanolamine-co-sebacate) (PPMS) was synthesized using 15-pentadecanlactone (PDL), MDEA and diethyl sebacate (DES). Other series of PACEs were designed by replacing DES with sebacic acid (SA) and by conjugating PEG to PPMS. These PACEs were used to form either polyplex or solid nanoparticles depending on the PDL content for nucleic acid delivery. PACEs with a low PDL content were formulated into polyplexes, and the vector was subsequently optimized for the type of nucleic acid to be delivered and the cell to be targeted. The sizes and morphologies of the nanoformulations were uniform and had transfection efficiencies comparable to commercially available delivery systems while being more biocompatible and biodegradable [85].

It is important to maintain a balance between mRNA binding strength with PACE and the ease of release when optimizing the transfection efficiency. A library of PACEs with different molecular weights was designed, and it was shown that polymeric chains shorter than 2 kDa are not sufficient to maintain a stable interaction with mRNA. On the other hand, chains with molecular weights of 10 or 20 kDa do not release mRNA at a sufficiently high rate. Based on a detailed evaluation, 5 kDa was proposed as the optimum molecular weight for efficient mRNA binding and release [84].

PEGylated-PACE systems have also been used as nucleic acid vectors. Two series of PACEs were synthesized using lipase-catalysed polymerization. In the first series, PDL, MDET, and SA were polymerized, whereas in the second series, PDL, DES, MDET, and PEG (5 kDa) were utilized for polymerization (PACE-PEG) [83,84,85]. To study the impact of PEGylation on transfection efficiency, PACE and variable percentages of PACE-PEG were mixed in the presence of different nucleic acids; thus leading to the formation of polyplexes with variable percentages of surface PEGylation [84]. siRNA-loaded polyplexes were found to have spherical morphologies. To measure the in vivo transfection efficiency, firefly luciferase mRNA-loaded nanoformulations were administered by intravenous injection and also locally in the lung. The luminescence measurements showed that mixing PACE with 5% PEG-PACE improved transfection efficiency to about 50% compared with PACE alone after local delivery but had no impact on delivery by intravenous injection, demonstrating that PEGylation efficacy depends not only on the PEG:PACE ratio but also on the administration route. Further analysis of transfection efficiency using different nucleic acid-loaded polyplexes revealed a threshold PEG-PACE concentration beyond which a further increase reduced the cellular uptake due to decreasing the positive charge density and electrostatic interaction with the cell membrane. The inhibitory concentration (IC) was nucleic acid-dependent, and mixing PEG-PACE with PACE increased the transfection efficiency at concentrations below the IC. At low PACE-PEG concentrations of up to 0.05%, the transfection efficiency for pDNA increased. The inhibitory concentration for pDNA was 0.1%, whereas those for mRNA and siRNA were 0.5% and 1%, respectively [84].

#### 3.1.6. Cationic Polyacrylates

Cationic polyacrylates are synthesized by modifying their side chains with ionizable amine groups. Poly N-dimethyl amino ethyl methacrylate (PDMAEMA) is the most common polyacrylate used for gene delivery, as it can be protonated relatively easily in a biological media due to the presence of tertiary amines with pKa of ~7.5, and it has a sponge effect in endosomes. Polyacrylates can be synthesized using highly efficient ATRP and RAFT polymerization methods, and an optimal co-monomer ratio can be utilized for tailoring mRNA loading efficiency [118]. It is feasible to have sufficient cationic segments for mRNA loading and hydrophobic ones for maintaining vector stability in biological media. Ionizable and protonated amine groups in cationic polyacrylates lead to an increase in cellular uptake and promote mRNA release within the cytosol through the deprotonation of amine groups [119,120]. PDMAEMA undergoes slow self-degradation and releases mRNA and non-toxic acrylic acid-based by-products. The comb- and sunflower-like architectures of HEMA-DMAEMA polymers have been synthesized and investigated for their transfection efficiency in T cells. These polymers were able to transfect primary T cells with mRNA molecules with 25% efficiency and a cell viability of ≥75% [120].

In another study, block copolymers comprising PEG and alkylated methyl methacrylate (MMA) and dimethyl amino methyl methacrylate (DMAEMA)/diethylamino methacrylate (DEAEMA) blocks were prepared. These polymers were self-assembled in such a way that, in mildly acidic environments, positively charged polyacrylates electrostatically interact with the mRNA to form a core along with hydrophobic MMA while PEG chains provide a hydrophilic shell. The influence of a variety of parameters, including the hydrophobic-to-cation segment ratio, MMA side chain length, molecular weight of the amphiphilic block copolymer, amine-based monomer type, and the hydrophobic: hydrophilic ratio of the polymeric chains on mRNA encapsulation, cellular uptake, transfection efficiency, and cytotoxicity were explored. It was noted that the DEAMEA-co-BMA-based polymers had the least cytotoxicity after 18 h of dosing, and the polymers with the lowest molecular weight (10,250 g/mol) had 6 times higher transfection efficiency than that of PEI-based systems [121].

A triblock copolymer containing poly (ethylene glycol) methyl ether methacrylate (PEGMA) for enhancing the stability of polyplex, DMAEMA for complexing with mRNA, and DEAEMA-co-BMA for inducing the sponge effect in endosomes was synthesized. A diverse sequential addition of monomers was used to explore their influence on mRNA transfection efficiency. The in vitro transfection efficiency of mRNA was analysed based on the GFP expression level. Polymers with PEGMA fragments in the center were highly stable and had 4–8 times and 4 times higher transfection efficiency values in RAW 264.7 and DC2.4 cells, respectively. The latter resulted in higher T cell-induced responses [122]. Polymers with no PEGMA block or a PEGMA block on one end had 3–25 times lower transfection efficiency values compared with that of lipofectamine, whereas polymers with a PEGMA block in the center provided the efficient transfection of both cell lines.

#### 3.1.7. Other Cationic Polymers

In addition to designing synthetic polymers for mRNA delivery, there has been an equally strong effort to utilize naturally occurring cationic polymers as mRNA vectors. Natural polymers with biodegradable backbones have great potential, and poly amino acids and polysaccharides with ionizable amine moieties and tailorable structures are commonly used for gene therapy.

##### Poly Amino Acids

Poly (amino acids), the so-called poly peptides, are synthetic or naturally occurring polymers with a wide variety of lengths and sequences. They contain positively charged segments including L-lysine, arginine, and histidine that establish electrostatic interactions with mRNA [123]. The mRNA transfection efficiency can be tuned by manipulating the type, number, and sequence of amino acids [124]. Poly L-lysine is often used in gene therapy. As with PEIs, its cytotoxicity depends on the molecular weight, and it is copolymerized with other monomers to modify its positive charge density. A dendronized polymer based on L-lysine dendrons and a di-cysteine backbone has been reported in which the disulfide bond in the backbone facilitated the release of mRNA through a redox mechanism [124]. L-lysine dendrons were functionalized with tryptophan and histidine to enhance cellular uptake and the sponge effect. The most efficient mRNA transfection was achieved using the PEGylated second-generation (G2) L-lysine dendron with a 2:1 ratio of histidine: tryptophan [125].

##### Poly Saccharides

Polysaccharides or glycopolymers are extraordinary constituents of biological systems that are used to improve the biocompatibility of gene delivery vectors. Chitosan and poly (glycoamidoamine) are examples of glycopolymers that are used for mRNA delivery. Chitosan is a deacetylated chitin, and its transfection efficiency and cytotoxicity are highly dependent on its deacetylated content. An injectable thermo-sensitive chitosan-based hydrogel with mRNA- and siRNA-loaded lipid nanoparticles for pancreatic cancer immunotherapy has been reported [126]. In another study, the transfection efficiency of chitosan-based mRNA vectors improved by ~75% in comparison with that of the lipid control when 2-propylacrylic acid was used as an excipient to form a triple-component formulation of PPAA/CS/mRNA (PPAA, poly (2-propylacrylic acid); CS, chitosan). The optimized nanoparticle with the CS/PPAA, N/P, and mRNA concentrations of 0.125, 5, and 450 μg/mL, respectively, yielded an mRNA expression level of 86% with no significant toxicity [95]. The mechanism for the increased transfection efficiency is uncertain; however, it was proposed to result from the endosomolytic activity of PPAA in acidic endosomes or the mRNA release induced by the competitive binding of PPAA to CS in the cytosol [95].

### 3.2. Non-Cationic Polymers

**Figure 9:**
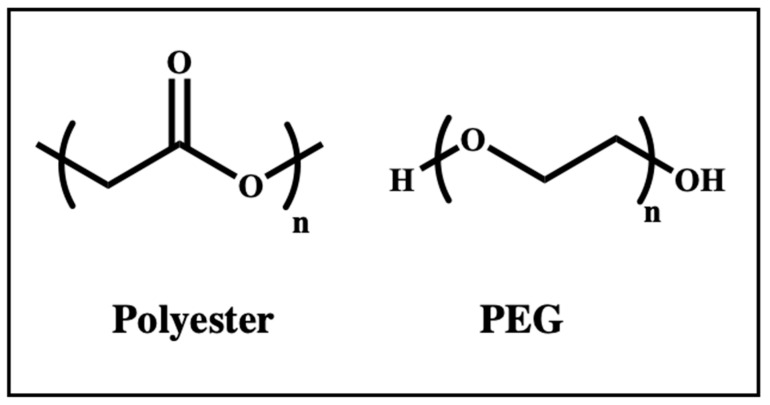
Chemical structures of non-cationic polymers for mRNA delivery as follows: that of polyester and that of polyethylene glycol (PEG).

Non-cationic polymers (Figure 9) lack positively charged centers, and their advantageous features as vectors for mRNA delivery include biodegradability, biocompatibility, as well as lower cytotoxicity. They are used mainly as core- or shell-stabilizing agents for mRNA and positively charged segments [127]. Two well-established non-cationic polymers for mRNA delivery are briefly elaborated below.

#### 3.2.1. Polyethylene Glycol (PEG)

PEG has been widely utilized in drug delivery either as a homopolymer or copolymer. It can help protect nucleotide cargo from degradation by ribonucleases and detection by the immune system. It forms a hydrated layer that prevents negatively charged serum proteins from attaching to the surface of the vector. PEG can also weaken the interaction of vectors with the negatively charged cell membrane, since it reduces the surface positive charge density. Consequently, the PEG/polymer ratio needs to be optimized [128]. The influence of PEG molecular weight on the transfection efficiency and cellular uptake of the mRNA-loaded polymeric nanoparticles has been evaluated. It was noted that although vectors coated with PEG_10000_ were less cytotoxic, they were unable to enter cells, whereas those prepared with PEG_2000_ had a higher cellular uptake [121]. This suggests that both the PEG/polymer ratio and the administration route should be optimized. Poly aspartic acid substituted with diethylenetriamine were designed for mRNA delivery to the lung and muscles. The expression of mRNA was optimal with 1:1 and 10:1 ratios of PEG: polymer after intravenous and intramuscular injections, respectively [88].

#### 3.2.2. Polyester

Biodegradable polyesters have attracted interest for use in gene therapy applications. They are hydrolysed in biological media to non-toxic products that can be cleared through the renal filtration. Polylactic acid (PLA), polyglycolic acid (PGA), poly (D, L-lactide-co-glycolide) (PLGA), polycaprolactone (PCL), and poly (β-hydroxybutyric acid) are common polyesters used for nucleic acid delivery [129]. They are generally synthesized through polycondensation or ring-opening polymerization. Although these polymers are safe, they suffer from an insufficient level of interaction with mRNA. Therefore, it is essential to modify the side chains with amine-based moieties, which preserve biodegradability and increase transfection efficiency. Functionalized trimethylolpropane allyl ether-co-suberoyl chloride polyesters were designed for luciferase-encoded mRNA delivery to lung cells through intravenous injection. The polymers were prepared through the condensation of trimethylolpropane allyl ether and diacyl chlorides. Varied backbones with different ratios of alkyl thiols and amine thiols were prepared for the post-modification of side chains. Using this approach, 5% F127 formulated polyester (PE4K-A17-0.33C12; PE4K, polyester with the molecular weight of 4200 g/mol; A17, 2-mercaptoethan ammonium chloride; C12, 1-dodecan thiol) was identified with the highest level of luciferase expression by screening for cells [91].

### 3.3. Stimuli-Responsive Polymers

Releasing nucleic acids from polymeric nanoparticles is another critical determinant of their efficiency. Diffusion, degradation of polymeric systems, and osmosis-induced cargo release are some of the common mechanisms of cargo release. There has been much effort devoted to incorporating stimulus-responsive moieties into the structure of delivery systems that enable controlled release at the desired site. The type of stimulus-response is adjusted according to specific needs and may utilize endogenous or exogenous stimuli. Internal stimuli are based on changes occurring around disease sites in the body and include pH, redox state, and enzyme activities, while external ones require the use of light, ultrasound, a magnetic field, etc. Polymers can be functionalized with a variety of stimuli-responsive chemical entities to impart single-stimulus or multi-stimuli responsivity [130,131,132].

#### 3.3.1. pH-Responsive Polymers

**Figure 10:**
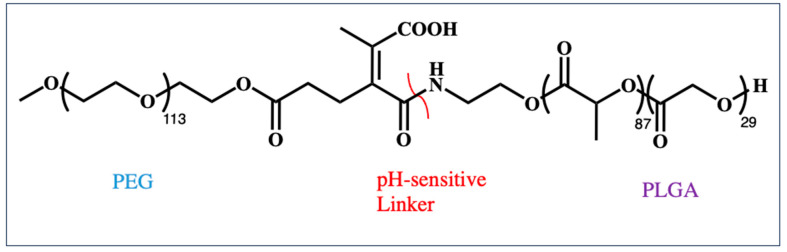
Structure of the MeO-PEG pH-sensitive linker PLGA polymeric chain, PLGA = poly (D, L-lactide-co-glycolide) [89].

Such polymers generally incorporate carboxylic acid and amine groups that can be easily deprotonated or protonated in biological media. A wide variety of pH-responsive polymers have been investigated to enhance the stability of polyplexes and their escape from mildly acidic endosomes. For instance, polymeric micelles that have a pH-sensitive cross-linked core consisting of cis-aconitic-modified PEG-PLL were designed for mRNA delivery. Unmodified PLLs were used to form poly-ion complexes with mRNA, and the stimuli-responsive PLLs had pH-sensitive amide bonds that could mediate hydrogen bonding and increase the stability of micelles at a neutral pH in the extracellular matrix. Amide bonds are broken within endosomes, thereby facilitating mRNA release through the sponge effect [90]. By using pH-sensitive linkages that have a tuneable pKa, it is possible to exploit moieties with opposing effects to enhance mRNA transfection efficiency. For example, PEG enhances in vivo circulation times and stability but reduces cellular uptake. It has been reported that mRNA-loaded nanoparticles composed of cationic lipids interacting with mRNA can be loaded into the PEG-PLGA nanoparticles with pH-sensitive linker (Figure 10) [89]. The rationale for this design was that it promoted accumulation around tumours through the EPR (enhanced permeability and retention) effect. PEG could be subsequently removed from nanoparticles through the cleavage of the pH-sensitive linker in the extracellular matrix surrounding the tumour cells, thereby increasing the cellular uptake of the nanoparticles. Using this design, mRNA encoding PTEN was delivered to breast cancer cells that were resistant to trastuzumab, thereby blocking the PI3K/Akt signalling pathway responsible for cell growth and proliferation [89].

#### 3.3.2. Redox-Responsive Polymers

Tuning mRNA release within the cell is as significant as polyplex stability in the extracellular space. The reducing environment of the cytoplasm due to the high concentration of glutathione (GSH) provides a rationale for designing redox-sensitive polymers that can release mRNA upon cell entry. Redox-responsive polymers generally contain disulfide bonds that are susceptible to cleavage by intracellular GSH [133]. The latter facilitates mRNA release and additionally helps reduce their toxicity due to the biodegradability of the polymers. In one study, poly(disulfide)-based systems were designed through the ring-opening polymerization of two disulfide-based monomers, one containing diethylenetriamine and the other with guanidyl units on their side chains. The resulting polymer had the ability to deliver CRISPR-Cas9-based genome editing machinery including mRNA, pDNA, and protein. The presence of disulfide bonds in the main chain led to the slow release of cargo over time. The flow cytometry results using 293T cells transfected with GFP mRNA showed higher cellular uptake and mRNA expression efficiency, 1.5 and 6-fold compared with those of lipofectamine 3000 and PEI, respectively. It was mediated by thiol-disulfide exchange with the cell membrane. The thiols on the surface of the nanoparticles are exchanged with the disulfide bonds existing within the structure of the membrane proteins. In addition, the reduction of the disulfide bonds by glutathione leads to rapid degradation [134]. In another study, a dual redox/temperature-responsive polymeric micelle system was prepared in which thiol was incorporated into poly (L-lysine), and a mixture of the PEG- and PNIPAM-modified poly (L-lysine) was prepared. This resulted in a di-sulfide-linked PLL core complexed with mRNA which had two different shells acting as protecting layers for mRNA. PNIPAM became hydrophobic in the biological environment; thus, creating a protective spatial barrier [96].

### 3.4. Polymer–Lipid Hybrid Systems

One of the most common approaches in designing polymer–lipid hybrid systems involves the interactions of phospholipids with mRNA and loading these into the polymer-driven nanoformulation with phospholipids on the polymer surface. Polymers that interact with mRNA can also be loaded into lipid formulations. Such systems exploit both polymers and lipids to form a highly efficient mRNA vector [135]. Due to the compatibility of lipids in the nanoparticle with the cell membrane, such lipid-based systems tend to fuse with the cell membrane more easily, leading to increased cellular uptake, endosomal escape, and overall transfection efficiency [136]. Polymers provide opportunities to introduce a variety of functionalities and add structural stability to avoid disassembly upon dilution in biological environments.

As an example of such polymer-lipid systems, zwitterionic phospholipidated cationic polymers were prepared to deliver mRNA to spleen and lymph nodes. Different alkylated dioxaphospholane oxide molecules were used to post-modify the cationic amine-based polymers [94]. The goal was to reduce cytotoxicity by decreasing the positive charge density and incorporate alkyl chains into the whole structure to increase the stability of the vector. To achieve this, glycidyl methacrylate was first polymerized using RAFT (reversible addition−fragmentation chain-transfer) polymerization, resulting in a polymer with epoxide side chains. Different amines were then conjugated with the polymer through a ring-opening reaction of the epoxide side chain to form cationic polymers. The resulting polymers underwent further reaction with alkylated dioxaphospholane oxide molecules to form zwitterionic polymer–lipid systems. The hybrid system had negative zeta potential, higher stability in the presence of serum proteins, a 39,500-fold enhanced mRNA transfection efficiency relative to the unmodified cationic polymers, better lipid fusion, and improved endosomal escape [94].

A charge-altering releasable transporter (CART) is also a combination of lipids with polymers that consist of oligo carbonate-*b*-alpha amino esters with a pH-dependent charge. A CART initially has a positive charge, which allows for efficient mRNA loading. Upon entering the cell, their positive charge density decreases due to neutralization and intramolecular arrangement that weakens the mRNA–vector interaction and facilitates mRNA release. Modification of lipids is necessary for optimal delivery, and long lipids provide better cellular uptake. Lipid-based vectors are preferentially accumulated in the liver after intravenous injection; however, lipid composition can be altered for targeting mRNA to the lymph nodes. For example, the FTY720-modified CART, in which FTY720 is a fingolimod sphingosine-1-phosphate receptor modulator, leads the mRNA vector towards S1P1, which expresses B and T cells to increase the activity of immune cells. The CART was synthesized through the ring-opening polymerization of *N*-boc morpholinone and MTC-dodecyl carbonate monomers, which was initiated by FTY720. Flow cytometry analysis demonstrated that FTY720-conjugated CARTs had an 80% higher mRNA expression efficiency in splenocytes compared with that of non-conjugated ones [93].

## 4. Conclusions

The power of mRNA-based therapeutics has been well demonstrated through the fast development of mRNA vaccines against COVID-19. It has expanded interest in utilizing mRNA for other diseases, and much effort has been devoted to improving the stability of mRNA in vivo. However, the design of vectors that protect mRNA and efficiently deliver it to the desired sites remains a major challenge. Recent developments in synthetic polymer chemistry suggest that polymer-based nanoparticles have great potential for mRNA delivery. Here, a brief overview of various non-viral mRNA vectors and their successe and limitations was developed. It focussed on the polymeric systems that have been most extensively explored and discussed strategies for improving their performance.

Considering the immense body of work on polymeric nanoparticles (largely polyplexes), further optimization of nanocarriers for mRNA will need to balance various nanoformulation parameters. The architectural complexity of macromolecule carriers has evolved, and the latest developments can be exploited when expanding polymer-based nanocarriers for gene delivery. Highly versatile polymers can be designed for optimizing the composition, morphology, and functionalization of nanoparticles. It can help design colloidal nanoformulations with desired characteristics including stimuli-responsivity and cell targeting. For example, branched star polymers, which are often referred to as miktoarm (mikto, Greek, meaning different) polymers, are utilized for drug delivery and could offer a platform for gene delivery. Their unique arm-by-arm construction on a central core allows for tunability in the spatial distribution of desired functions for optimization of overall efficacy, cytotoxicity, as well as targeting. Core–corona-based micellar nanoparticles are being explored for use in gene therapy, and it is now well documented that branched star polymers can help to fine-tune the properties of such nanoparticles. Aqueous nanoformulations from miktoarm stars have a much denser corona, which can better protect the nucleic acid cargo, lower the critical micelle concentration (increasing stability), and controlled cargo release. Considering the composition of the branched star polymers can be adjusted through number of arms in the polymeric architecture, their nanoformulations can be tailored for optimal properties and offer a promising direction for advances in mRNA delivery.

## Figures and Tables

**Table 2:** Summary of polymeric systems used for mRNA delivery.

Polymer	Molecular Weight of mRNA-Interacting Polymer	N:P Ratio	NP Formation Method	Characterization Methods	Reference
PEI-g-PEG	PEI (22 kDa)	8	Flash nanocomplexation Solvent: Water	DLS, Zeta Potential, TEM	[70]
F-PEI	1.8 kDa	*w*:*w* (1:1)	Direct mixing Solvent: Water	DLS, Zeta Potential, TEM	[76]
CD-PEI	bPEI (2 kDa)	16 (Optimized)	Direct mixing Solvent: Water	DLS, Zeta Potential, TEM	[77]
PBAE@PEG-lipid	PBAE (2354 Da)	50 (Optimized)	1. Pipette mixing or 2. Microfluidic mixing or microfluidic device mixing Dialysis method Solvents: PBAE in DMSO, PEG-lipid in ethanol, mRNA in sodium acetate buffer	DLS	[50]
PBAE NP-doped PEG-PBAE	-	*w*:*w* 25:1 and 50:1	Direct mixing, PBAE in DMSO, mRNA in water Both phases diluted in acetate buffer before mixing	DLS, SEM	[51]
PNP (PEG-PLGA@Lipid-PBAE	PBAE (4137 Da)	-	Double emulsion–solvent evaporation Solvent: polymers in DCM, mRNA in water	DLS, Zeta Potential, TEM	[61]
PBAE-co-PCL terpolymer	850–2800 Da	50	Direct mixing Polymer in DMSO diluted in acetate buffer, mRNA in acetate buffer	DLS, Zeta potential, Cryo TEM	[78]
Oligopeptide end modified PBAE	-	25	Direct mixing Solvent: acetate buffer	DLS, NTA, Zeta potential	[79]
hPBAE	20 kDa	50	Direct mixing Solvent: acetate buffer	DLS, Zeta potential, TEM	[80]
PBAE terpolymer@PEG-Lipid	2376 Da	57	Direct mixing Solvent: pH = 5.2 buffer	DLS, Cryo TEM	[81]
MDET@Cp	4760–5050 Da	55	Direct mixing Solvent: polymer in DMSO diluted in acetate buffer+ mRNA	DLS, Zeta potential	[82]
PACE terpolymer	2–20 kDa	100	Direct mixing Solvent: polymer in DMSO diluted in acetate buffer, mRNA in acetate buffer	DLS, Zeta potential	[83]
PACE@PEG	17,500 Da	100	Direct mixing Solvent: polymer in DMSO diluted in acetate buffer, mRNA in acetate buffer	DLS, Zeta Potential, TEM	[84]
PACE	7 kDa	100	1. Polyplex formation: direct mixing Solvent: polymer in DMSO diluted in acetate buffer, mRNA in Acetate buffer 2. Solid NP formation: single or double emulsion-solvent evaporation method Polymer in DCM, mRNA in acetate buffer, PVA	DLS, Zeta Potential, TEM, SEM	[85]
PAMAM@PEG-lipid	-	5.22	Microfluidic device, dialysis, polymer and PEG-lipid in ethanol, mRNA in citrate buffer pH = 3	DLS, TEM	[86]
Histidine rich Peptide	-	4	Direct mixing, Solvent: Opti-MEM	DLS, Zeta potential	[87]
(DET)-P(Asp)@PEG	18,747 Da	32	Direct mixing, Solvent: HEPES	DLS, Zeta potential	[88]
Meo-PEG-Dlinkm-PLGA	Total polymer: 16 kDa	-	Nanoprecipitation, Polymer and G0-C14 in DMF, mRNA in water	DLS, Zeta potential, TEM	[89]
PEG-pLL (CAA)	6725 Da	4	Direct mixing, Solvent: HEPES	DLS	[90]
PE4K-A17 0.33C12	-	30	Direct mixing, Solvent: Polymer in DMSO, mRNA in citrate buffer	DLS, Zeta potential	[91]
PEG-PAsp (DET)-Chol (+) @Chol (+)-OligoRNA	-	3	Direct mixing, Solvent: HEPES buffer	DLS, Zeta potential, TEM	[92]
FTY720-CART	9700 Da	10	Direct mixing, Solvent: polymer in DMSO, mRNA in PBS buffer	DLS, Zeta potential	[93]
zwitterionic phospholipidated polymers	3 kDa	20	Dialysis Solvent: PEG-lipid in Ethanol, mRNA in citrate buffer Dialyzed with PBS	DLS, Zeta potential, TEM	[94]
CS/mRNA/PPAA	5 and 10 kDa	5	Direct mixing Solvent: CS, PPAA stock solutions in water or buffer, mRNA in buffer.	DLS, Zeta potential	[95]
cRGD-PEG@(PEG)-(PLys) (thiol)@(PNIPAM)-PLys(thiol)	8000	1.5	Direct mixing Solvent: HEPES buffer, dialysis	AFM, TEM, DLS, Zeta potential	[96]

Abbreviations: PEI, polyethyleneimine; PEG, polyethylene glycol; F-PEI, fluoroalkane-grafted PEI; CD-PEI, cyclodextrin-PEI conjugate; PLGA, poly (lactide-co-glycolide); MDET@Cp, N-methyldiethanolamine@alkyl diol; DET-P(Asp), diethylenetriamine-substituted poly aspartic acid; Dlink_m_, pH-liable linker; PLL (CAA), poly (trimethylolpropane allyl ether-co-suberoyl chloride); Chol (+)-OligoRNA, chol-tethered RNA oligonucleotides; FTY720-CART, fingolimod-conjugated charge-altering releasable transporter; PPAA, poly (2-propylacrylic acid); CS, chitosan; Plys, polylysine.
